# Organised Charity Abroad

**Published:** 1899-01-07

**Authors:** 


### ORGANISED CHARITY ABROAD.

### THE HOUSE OF THE BON SAUVEUR, AT CAEN.—II.

Anne Le Roy, the originator of the Congregation of the Bon Sauveur, daughter of a cooper and little shopkeeper in the Rue Saint Jean, was born in 1691. She had a strong leaning towards the life of an active Sister of Mercy, and wished to join the Ursulines, but could not afford the necessary dowry. She therefore went to help in a community at Saint Lo called the Bon Sauveur. Here she was quite in her element, but was forced to withdraw and return to Caen on account of ill-health. She then took a small house in the parish of Saint Michel de Vaucelles, with her friend Mademoiselle Lecouvreur, and began to teach little children and to visit the sick. In 1725 they were joined by four others. M. de Creully, Superior of the Seminary of Caen, consecrated a room in their house as a chapel where the parish clergy could say Mass, and four years later Anne Le Roy was nominated Superioress of the little community. A larger house became indispensable, and one was taken in the Ange, containing kitchen, refectory, class-rooms, dormitories, and chapel. The Bishop of Bayeux gave the new community the Rule of the Bon Sauveur of Saint Lo, and they received Royal Letters Patent in 1739. It may not be out of place to give a rough translation of the statutes of the community. These are 12 in number: (1) The objects of this society—(*a*) To take charge of the insane; (*b*) to educate girls; (*c*) to visit the poor; (*d*) to keep a free dispensary; (*e*) to have poor-schools; (*f*) to provide a retreat for the aged; (*g*) to train school-mistresses. (2) House to be governed by a Superioress, and Assistant-Superioress, and three advisers. (3) The Superioress to be elected every three years. (4) The Superioress, in concert with the Bishop, nominates the Assistant-Superioress, the housekeeper, and the three advisers. (5) The Superior allots work to Sisters at discretion. (6) Lay Sisters act as servants. (7) In things spiritual the Sisters are subject to the Bishop, in things temporal to the magistrate. (8) Each Sister (admitted into the society) is received by the Superioress and Sisters. (9) The probation is two years and three months. (10) Each Sister retains possession of her property, or of property which may devolve to her after joining the society. The community has only the usufruct. (11) The community has the right to expel any member for grave cause should such case arise. The expelled Sister shall have no right to compensation. (12) These statutes are the only ones recognised by the Congregation. During the Revolution and the Wars of Napoleon the new community went through many and varied experiences. A certain proportion of the Sisters remained in their quarters in charge of the insane, but the greater number were driven out, and were forced to live as they could in various houses of the town. The Capuchins were likewise expelled, and the Sisters were able to [illegible]btain the old Capuchin convent for the Bon Sauveur. This convent, which was built in 1576, stands in part of the fief de Brucourt, a dependency of the Abbaye-[illegible]x-hommes given to the Capuchins by Cardinal [illegible]arnese when Abbot of Saint Etienne. Incorporated with the new buildings, the old quadrangle, with the round well in its centre, forms the nucleus of the modern convent. In May, 1805, M. Jamet succeeded in collecting the scattered community. Portalis sent a report on the Bon Sauveur to the First Consul, who approved it, and his decree—5th Ventose an XII.—fixes its standing as an institution.

After Waterloo the soldiery were quartered at Caen, and the Sisters had to give up their chapel refectory and community rooms. Shortly after this Napoleon nominated Senator Comte de Maubourg Governor of Caen. The prosperity of the Bon Sauveur dates from this appointment, for he took a personal interest in the community, and was a friend of M. Jamet's. At this time, however, the year of Napoleon's banishment to St. Helena, Caen was visited by typhus fever, which raged for ten years.

In 1818 the insane and the deaf and dumb were moved from Beaulieu to the Bon Sauveur, and the Préfet and Corporation of Caen arranged with M. Jamet to entrust all the insane of the Department of Calvados to his congregation. At the same time, the Consul-Général made a grant of 50,000 francs for a house to hold them. Paris and other Departments have the right to send a certain number of patients as there is room to receive them. The Sisters also take private cases, who, of course, pay handsomely. During the heats, August-September, 1898, a party of fifty insane arrived from Paris in one week, distracted by the temperature of the capital.

The Bon Sauveur has three affiliated houses—at Bégars (Côtes-du-Nord), at Albi (Tarn), and at Pont-l'Abbé (La Manche). In January, 1894, the Mother House at Caen had 983 patients from the Departments, and 883 private cases altogether. The grounds contained 2,500 persons. The house is not under a "regiment" of women, as may appear from the above, for, besides the three chaplains, who are ever ready with advice, there is a Superior-general, who has equal authority with the Superioress, and who is always a man of culture and position. M. Goudier, Vicar-general of the diocese of Bayeux, is the present Superior. Jean François Desprez, for 40 years Proviseur du Lycée de Caen, was his predecessor, who succeeded the first Superior, Pierre François Jamet "fondateur de l'Ecole des Sourds-muets, Chanoine Honoraire de Bayeux, d'Albi et de Coutances, ancien Recteur de l'Académie de Caen, membre des Academiés de Caen et de Rouen, Chevalier de la Légion d'Honneur," born at Fresnes, 1762; died at Caen, 1845. "Laudent eum opera ejus" is the motto placed on his tomb in the little chapel erected to his memory in the grounds of the Bon Sauveur.

